# Biocide leaching during field experiments on treated articles

**DOI:** 10.1186/s12302-016-0074-9

**Published:** 2016-02-20

**Authors:** Ute Schoknecht, Helena Mathies, Robby Wegner

**Affiliations:** 1 Bundesanstalt für Materialforschung und -prüfung (BAM), Berlin, Germany; 2 MPA Eberswalde, Materialprüfanstalt Brandenburg GmbH, Eberswalde, Germany

**Keywords:** Biocidal products, Treated articles, Emission, Leaching, Field leaching test, Paint, Impregnated textile, European regulation

## Abstract

**Background:**

Biocidal products can be sources of active substances in surface waters caused by weathering of treated articles. Marketing and use of biocidal products can be limited according to the European Biocidal Products Regulation if unacceptable risks to the environment are expected. Leaching of active substances from treated articles was observed in field experiments to obtain information on leaching processes and investigate the suitability of a proposed test method.

**Results:**

Leaching under weathering conditions proceeds discontinuously and tends to decrease with duration of exposure. It does not only mainly depend on the availability of water but is also controlled by transport processes within the materials and stability of the observed substances. Runoff amount proved to be a suitable basis to compare results from different experiments. Concentrations of substances are higher in runoff collected from vertical surfaces compared to horizontal ones, whereas the leached amounts per surface area are higher from horizontal surfaces. Gaps in mass balances indicate that additional processes such as degradation and evaporation may be relevant to the fate of active substances in treated articles. Leached amounts of substances were considerably higher when the materials were exposed to intermittent water contact under laboratory conditions as compared to weathering of vertically exposed surfaces.

**Conclusions:**

Experiences from the field experiments were used to define parameters of a procedure that is now provided to fulfil the requirements of the Biocidal Products Regulation. The experiments confirmed that the amount of water which is in contact with exposed surfaces is the crucial parameter determining leaching of substances.

## Background

Recent studies demonstrated that the application of material preservatives can cause input of active substances into surface waters. Wittmer et al. [1] studied the dynamics of selected biocides and pesticides in catchments that represent different types of land use. The observed concentration patterns indicate that urban areas can be sources of biocides in water samples. Burkhardt et al. detected active substances that were leached from façades and bitumen membranes at different sampling sites in a separate sewer network in Zurich [2], and described the leaching dynamics of active substances from façade renders that were applied to a model house [3]. Evidence of active substances in a suburban stormwater catchment was related to the buildings in the investigated Danish settlement by Bollmann et al. [4]. Gromaire et al. [5] investigated runoff from different roof materials that were cleaned using biocidal products. The concentration of the active substance increased in runoff even 1 year after its application. Occurrence of biocides in stormwater in Berlin was related to the actual urban structure by Wicke et al. [6]. Increased amounts of carbendazim, diuron, terbutryn and mecoprop in stormwater were observed in an area with a large number of recently renovated buildings and therefore attributed to construction materials.

This input of biocides into water bodies can be caused by leaching processes if treated articles are exposed to precipitation. Substances from the materials can reach runoff water and can be transported into surface water.

Marketing and use of biocidal products can be limited according to the European Biocidal Products Regulation (BPR) [7] if unacceptable risks to the environment are expected. However, restrictions depend on reliable knowledge on the expected release into the environment.

Leaching processes from treated articles were investigated both under laboratory and field conditions during a research project that was initiated and co-financed by the Federal Environment Agency. One of the project tasks was to develop and prove the suitability of test methods that can be provided as guidance for producers in order to support data provision for the authorization of biocidal products. It was also intended to further develop mathematical modelling of leaching data to support the development of harmonized assessment procedures.

During this project, seventeen different treated articles were investigated under laboratory conditions. Test specimens of six different paints and pieces of an impregnated textile were exposed to natural weathering at three different locations. Experiments on one of the paints were started three times at intervals of 3 months on the same location in order to investigate initial phases of leaching processes under different meteorological conditions. Test specimens from another paint and the impregnated textile were horizontally and vertically oriented (see Table 1 for the experiments that are considered in this presentation). Data on four active substances with different chemical structures and physico-chemical characteristics were obtained from runoff samples (see Table 2).

**Table 1:** Overview on field experiments

Treated article		Experiments				
Name	Initial amount of a.s.^a^ [mg/m^2^]	Test site	Orientation	Code	Start	Duration [months]
Paint A on wood	Diuron, 250 OIT, 130 Terbutryn, 250	MPA Eberswalde	Vertical		29.05.2012	25
Paint B on wood	Diuron, 390 OIT, 200 Terbutryn, 390	MPA Eberswalde	Vertical	M-1a	29.05.2012	25
		MPA Eberswalde		M-1b	29.05.2012	25
		MPA 1 km outside Eberswalde^b^		M-1c	29.05.2012	18
		MPA Eberswalde		M-2	29.08.2012	22
		MPA Eberswalde		M-3	29.11.2012	19
		BAM Berlin		BAM	29.08.2013	23
Paint C on fibre cement	OIT, 300 Terbutryn, 560	MPA Eberswalde	Horizontal		29.05.2012	18
		BAM Berlin	Vertical		29.08.2013	23
Impregnated textile	Carbendazim, 700	MPA Eberswalde	Vertical^c^ and horizontal		29.08.2012	15
		BAM Berlin	Vertical (SSW)		29.08.2013	7

^a^Initial amounts of active substances were confirmed by chemical analysis of the treated articles

^b^Hill in open landscape (Drachenkopf)

^c^Test specimens of the impregnated textile were oriented towards SE, SW, NE and NW

**Table 2:** Active substances investigated during the field experiments

Active substance	CAS-No.	Function	Molar mass	Water solubility (pH 7)	Log K_OW_	Reference
			g/mol	mg/l		
Carbendazim	10605-21-7	Fungicide	191.2	8	1.51	[17]
Diuron	330-54-1	Algicide	233.1	37	2.85	[17]
OIT^a^	26530-20-1	Fungicide, bactericide	213.3	480	2.45	[18]
Terbutryn	886-50-0	Algicide	241.4	22	3.65	[17]

^a^2-Octylisothiazol-3(2H)-one

Data on three paints and the impregnated textile were selected to exemplary illustrate the main observations on leaching of biocides from treated articles during the field experiments. The complete dataset is presented in a research report [8].

## Results and discussion

### Precipitation—driving rain—runoff

Construction products and treated articles that are used outdoors are occasionally exposed to water contact. Exposure differs for horizontally and vertically exposed surfaces. Horizontal surfaces are exposed to the total amount of precipitation. However, a part of the water cannot be collected as runoff due to splashing and evaporation. Vertical surfaces are only exposed to a part of overall precipitation depending on wind direction and velocity that determine the amount of driving rain. Runoff from these surfaces is considerably lower. This is illustrated in Table 3 for test specimens of two selected types of materials. The amount of driving rain reaching the surface is additionally affected by building geometries in real structures. The retention period of water and therefore water uptake of the materials can also be different for real structures and small test specimens. This affects the ratio between runoff and driving rain. Small test specimens obtain higher amounts of driving rain than large surfaces. Comprehensive information on rainwater runoff from façades is given by Blocken et al. [9].

**Table 3:** Precipitation, driving rain towards the test specimens and runoff from the test specimens during field experiments at MPA Eberswalde

Treated article	Orientation	Duration	Precipitation	Driving rain	Runoff
		Months	mm	l/m^2^	l/m^2^
Paint A and paint B on wood	Vertical (SW)	25	1370	269	44
Paint C on fibre cement	Horizontal	18	1049		818
Impregnated textile	Vertical (SW)	15	749	127	23
	Horizontal	15	749		657

### Emission of active substances during rain periods

**Fig. 1:**
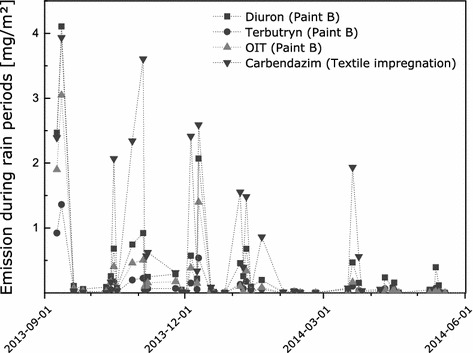
Emission of active substances from treated articles during rain events; data from BAM field experiments on paint B and an impregnated textile

Emission of active substances into runoff proved to be a discontinuous process. The emissions per surface area tend to decrease with time of exposure. Maximum and minimum emissions occurred simultaneously for all investigated active substances from different treated articles (see Fig. 1). There were a number of rain events that caused runoff from vertical surfaces but very low emission of active substances. However, these samples usually represent small runoff volumes.

**Fig. 2:**
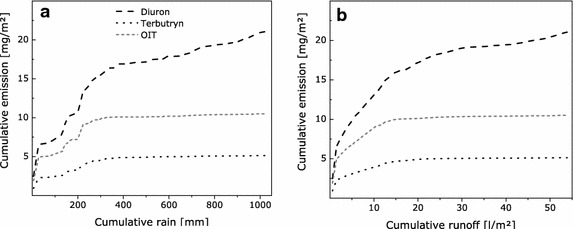
Emission curves for different active substances from paint B during 23 months of exposure at BAM related to cumulative rain (**a**) and runoff (**b**)

Figure 2 illustrates that emissions from vertical surfaces depend on runoff rather than the amount of rain. Emission curves related to the amount of runoff slightly deviate from regular curves, indicating that additional parameters may affect leaching processes. Emission of OIT almost stops after a certain period of time. It is assumed that this is caused by depletion of this substance in the outermost layers of the coating which can be caused by degradation and evaporation.

### Repeatability of field experiments

**Fig. 3:**
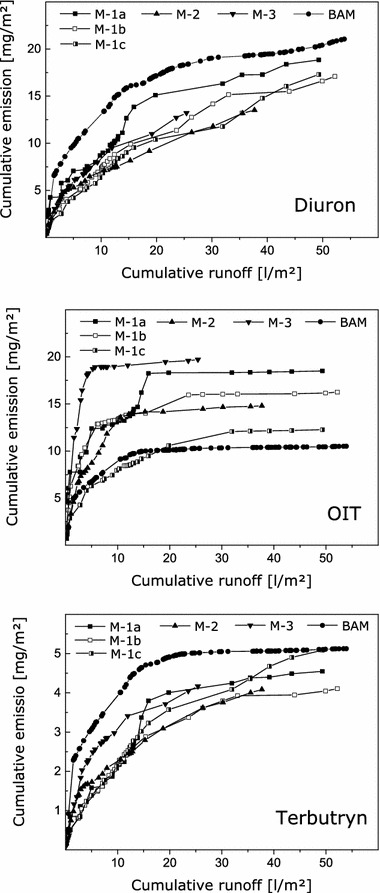
Emission curves for diuron, terbutryn and OIT from six experiments on paint B that were performed at three different locations and started at different times (see Table 1 for details on the experiments)

Emissions into runoff water were recorded for three active substances with different structures and physico-chemical properties (see Table 2) for six experiments on paint B (see Table 1 and Fig. 3). The amounts of active substances detected in runoff water during the whole periods of the experiments differed between 3.4 and 5.4 % of the original amount of diuron, between 5.3 and 9.9 % of the original amount of OIT, and between 1.0 and 1.3 % of the original amount of terbutryn during 18–25 months of outdoor exposure. Precision of the field experiments mainly depends on measurement uncertainty of the analytical methods (see “Methods”). Average uncertainty of the analytical results for the runoff samples is assumed to be about 5 %. Different emission curves are interpreted as actual differences in the leaching processes and are not related to measurement uncertainty.

Some differences were observed between the emission curves for single experiments on paint B. The emission curves are related to the volume of collected runoff water, i.e. similar exposure of the test specimens to runoff water. The observed differences indicate that the amount of leached substances does not only depend on the amount of runoff water but is affected by additional parameters. This is further supported by the fact that differences were not only observed for data on the same active substance from different experiments but also between curves for different active substances from the same test specimen. For instance, the initial emissions of terbutryn were higher during the experiment that was started at the end of November (M-3) compared to experiments that were started at the end of May (M-1a, b and c) and at the end of August (M-2). This effect is not visible for diuron. The large differences between emission curves for OIT are probably caused by different degrees of depletion. It is assumed that additional parameters affect transport processes of the substances to the material surfaces as well as losses due to degradation and evaporation of the active substances.

### Emissions depending on the orientation of test specimens

**Fig. 4:**
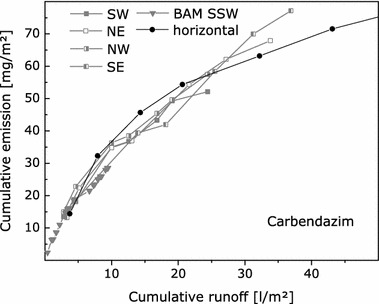
Emission curves for carbendazim from six experiments on the impregnated textile that was oriented to different directions and investigated at two different locations (see Table 1 for details on the experiments)

The emissions per surface area were higher for horizontally exposed test specimens compared to vertically exposed ones. Emissions of active substances were 26 mg/m^2^ OIT and 66 mg/m^2^ terbutryn of the horizontally exposed test specimen compared to 6.0 mg/m^2^ OIT and 8.6 mg/m^2^ terbutryn of the vertically exposed test specimen in the experiments on paint C. Higher emissions of carbendazim were also observed for a horizontally exposed test specimen compared to vertically exposed ones of the impregnated textile (see Table 4). However, concentrations of active substances in eluates were higher in runoff samples from vertically compared to horizontally exposed test specimens (see Table 5). In contrast to the observations from repeated experiments on paint B, the emission curves for carbendazim from the piece of impregnated textile strongly depend on the amount of runoff, irrespective of the orientation towards different directions, the test location and starting time of the experiment. Emissions from the horizontally exposed test specimen are also in line with the other emission curves (see Fig. 4). This is somewhat unexpected, since similar amounts of runoff were collected within about 1 month from the horizontally oriented test specimens compared to 15 months from the vertically exposed test specimens. That means that vertically oriented test specimens were much longer exposed to environmental influences, and degradation of carbendazim could occur. Probably, carbendazim is relatively stable towards degradation. This is supported by laboratory studies that indicate that carbendazim is stable towards artificial UV radiation in paints, renders and the impregnated textile [8, 10].

**Table 4:** Mass balances for active substances from field experiments at MPA Eberswalde

Treated article	Orientation	n	Active substance	Recovery of the original amount of active substance			
				Runoff	Treated article^a^	Wood	Gap in mass balance
				%	%	%	%
Paint A on wood	Vertical (SW)	1	Diuron	2.6	25	5	73
			OIT	1.7	20	10	79
			Terbutryn	0.3	8	0.3	92
Paint B on wood	Vertical (SW)	4^b^	Diuron	4.4	34	6	55
			OIT	4.7	12	14	70
			Terbutryn	1.3	27	0.7	71
Impregnated textile	Vertical	4^c^	Carbendazim	9.0	75		16
	Horizontal	1		46	40		14

*n* number of test specimens

^a^Data represent content in either paint layers and wood (sum) or the impregnated textile

^b^Data were obtained from experiments M-1a, M-1b, M-2 and M-3 (see Table 1)

^c^Data were obtained from experiments at MPA Eberswalde (see Table 1)

**Table 5:** Concentration ranges of active substances in runoff samples depending on the orientation of the test specimens

Treated article	Orientation	n	Duration of sampling^a^	Active substance	Concentration in runoff samples^b^		
					Minimum	Median	Maximum
			Months		µg/l	µg/l	µg/l
Paint C on fibre cement	Vertical	26	4.5	OIT	4	503	1991
				Terbutryn	9	354	1049
	Horizontal	24	4.5	OIT	1	14	2164
				Terbutryn	2	43	416
Impregnated textile	Vertical	70^c^	8	Carbendazim	22	2752	7545
	Horizontal	19			51	713	4288

*n* number of runoff samples

^a^Data represent single rain periods. Runoff samples from single rain periods were only analysed during the first few months of the experiment

^b^Data for horizontally exposed paint C on fibre cement and impregnated textile were obtained from experiments at MPA Eberswalde, data for vertically exposed paint C on fibre cement were obtained from the experiment at BAM (see Table 1)

^c^Data originate from four test specimens that were oriented to different directions (see Table 1)

### Mass balances

Mass balances were calculated for test specimens after outdoor exposure (see Table 4). Part of the active substances was transported from the coatings into the wooden substrate, probably depending on water solubility of the observed substances (see water solubility data in Table 2). The differences between the original amounts of active substances and the amounts detected in the runoff samples and the test specimens are high. This indicates that additional processes also control the fate of diuron, OIT and terbutryn. Evaporation and degradation are assumed to contribute to the fate and behaviour of these substances in the environment. In contrast, the original amount of carbendazim was almost completely detected in the runoff and the test specimens after outdoor exposure of the impregnated textile. Obviously, carbendazim in the piece of impregnated textile is less affected by competing processes. Leaching of carbendazim from pieces of the impregnated textile was relatively high. This was unexpected since leaching of carbendazim from paints and renders was observed to be relatively low, i.e. in the range of data for terbutryn [2, 10]. It was assumed that the textile impregnation itself is affected by water contact and UV radiation. Both effects were demonstrated in laboratory tests. Water repellency was decreasing, and water uptake was increasing after exposure to water, and leaching of carbendazim increased after artificial UV radiation of the impregnated textile [8].

### Comparison of emissions in laboratory and field experiments

**Fig. 5:**
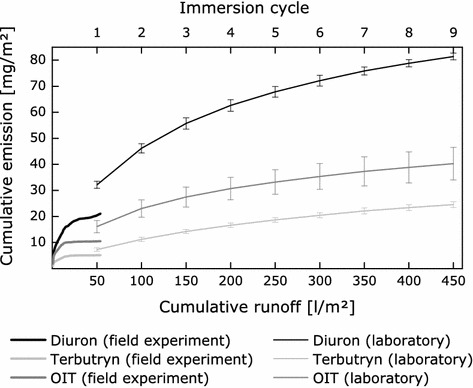
Comparison of emissions of diuron, terbutryn and OIT from paint B during field and laboratory experiments at BAM. Cumulative emissions observed during the laboratory test are related to the number of immersion cycles, each representing water contact to 50 l/m^2^ within two immersion periods of 60 min. The laboratory data represent mean values from four experiments, and the *error bars* indicate standard deviation. Cumulative emissions during the field experiment are related to the collected runoff

Leaching of active substances from the same treated articles was also investigated according to EN 16105 [11]. Test specimens are exposed to intermittent water contact during nine immersion cycles of 1-h immersion, 4-h drying and 1-h immersion within 3 weeks under controlled laboratory conditions. Altogether sixteen emission curves for four different active substances from six different paints and one impregnated textile from field experiments were compared with laboratory test results. Leaching of diuron, OIT and terbutryn from the investigated paints was considerably higher during the laboratory tests than that during 23 months of outdoor exposure. This is exemplary illustrated for paint B in Fig. 5.

**Fig. 6:**
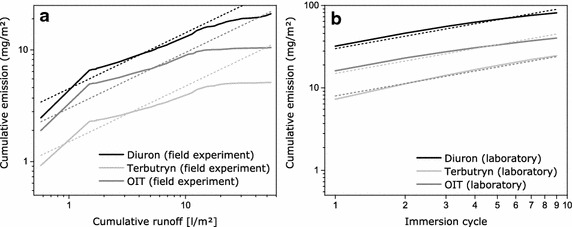
Double-logarithmic graphs of emission curves for diuron, terbutryn and OIT from paint B for **a** field and **b** laboratory experiments at BAM. The *dotted lines* represent lines with a slope of 0.5. These lines indicate diffusion-controlled processes. For explanation, diffusion proceeds proportional to the square root of time, i.e. diffusion ~ time^0.5^. The exponent 0.5 becomes factor 0.5 in logarithmic functions. It is assumed that the amount of runoff represents certain duration of water contact. The number of immersion cycle represents duration of water contact, i.e. each immersion cycle includes 2 h of water contact

Information on possible mechanisms that control leaching processes can be obtained from emission curves presented as double-logarithmic graphs (see Fig. 6). Leaching of diuron, OIT and terbutryn from paint B appears to be mainly controlled by diffusion in the laboratory test. In contrast, only certain periods of field experiments on paint B are controlled by diffusion processes. Solution of the active substances on the coating surface probably causes faster emission at the beginning of the field experiment, whereas the process slows down due to depletion of these substances on the surface after a period of mainly diffusion-controlled emission. Styszko et al. [12] investigated transport processes of active substances through acrylate and silicone renders. They concluded that transport depends on the availability of water, and diffusion is probably the dominant mechanism under the applied experimental conditions.

## Conclusions

Possible environmental impact of biocides that are applied in materials and exposed to weathering can only be assessed if leaching processes are understood. This requires data from appropriate tests. These tests should be harmonized, not only to define requirements but also to enable harmonized procedures to interpret test results.

The applied test procedure is based on the NT build 509 [13] procedure that was released for testing of preservative-treated timber by the Nordic Innovation Centre. The procedure became the basis for harmonization within CEN, resulting in the Technical Report CEN/TR 16663:2014 [14] that also refers to preservative-treated wood. The basic principle of this procedure was tested for treated articles that contain other biocidal products than wood preservatives (PT 8, as defined in the BPR) during the research project and proved to be suitable for coatings that include film preservatives (PT 7) and a textile including a PT 9 product (fibre, leather, rubber and polymerized materials preservative). Therefore, a guidance document [15] was prepared and adopted by European competent authorities together with guidance on a laboratory method [16] for testing of biocidal products that belong to PT 7, PT 9 and PT 10 (construction material preservatives).

The performed tests demonstrated that the amount of water that is in contact with the exposed surfaces is the crucial parameter that determines leaching of substances. The availability of water is not necessarily defined by the amount of precipitation but depends on driving rain in the case of vertically exposed surfaces. Consistency of emission curves is best if the results are related to the amount of runoff. Differences between emission curves from repeated experiments indicate influence of additional meteorological parameters. It is assumed that these parameters affect transport processes within the materials and losses due to degradation and evaporation. The observed gaps in mass balances also indicate considerable influence of competing processes on the fate and behaviour of the investigated substances.

Horizontally oriented surfaces are exposed to higher amounts of precipitation than vertically oriented ones. Therefore, total emissions from these surfaces are higher, whereas concentrations of substances in runoff can be higher from vertically exposed surfaces.

Emissions of substances can be considerably higher in laboratory tests based on intermittent water contact than in field experiments. Field experiments on vertically exposed test specimens can be considered to be conservative compared to practical conditions. This is caused by (1) the orientation of the test specimens towards directions that are exposed to high amounts of driving rain and (2) the use of relatively small test specimens. This causes relatively high amounts of contact water compared to materials in the intended use conditions.

## Methods

### Treated articles

Information on the investigated treated articles and active substances is given in Tables 1 and 2. The paints were all waterborne and either based on acrylates (paint A and C) or polymer dispersions (paint B). Paints A and B are the paints for wood, whereas paint C is a roof paint. Mixtures of active substances were added to commercially available paints by producers according to the requirements of this study. Therefore, liquid formulations were used although a number of modern paints now contain microencapsulated active substances. The textile was treated in an industrial procedure by the producer using an impregnation material that contains biocides. The contents of the active substances were confirmed by chemical analysis of the product.

### Preparation of test specimens

Paints A and B were applied to birch plywood boards (dimensions: 76 × 74 × 1 cm^3^). The side faces of the plywood boards were sealed using ‘Pyrotect Lack’. Paint C was applied to fibre cement boards (dimensions: 76 × 74 × 1 cm^3^). Paint C was also applied to the side faces of the fibre cement boards. All paints were applied according to the manufacturer’s instructions referring to the amount of paint per surface area, application of ground coats and the duration of drying phases. Paints dried at room temperature were protected from direct radiation for a period of 5 days until the field experiments were started. Textile samples were fixed to wooden frames of 76 × 74 cm lateral lengths.

### Leaching experiments

Field experiments were performed based on the procedure described in NT build 509 [13]. An overview on the field experiments is given in Table 1. Test specimens were installed at different test sites and oriented either vertically or horizontally. Runoff water was sampled at least once a week in case of rain events during this period. Each runoff sample was analysed for the active substances during the first 4.5–8 months of the experiments in Eberswalde and for the whole duration of the experiments performed at BAM. Later, aliquots of the runoff samples from the experiments performed at MPA Eberswalde were stored and combined for analysis. The samples were analysed according to the scheme given in NT build 509 [13]. One additional sample was analysed within the defined sampling periods. The amount of driving rain was measured at both test sites of MPA Eberswalde. Driving rain was collected in rain gauges that were installed immediately adjacent to the test specimens facing in the same direction. The amount of collected water was determined by weighing and related to the receiving surface area of the rain gauges. The test sites belong to areas that are only moderately affected by driving rain according to DIN 4108-3 [19].

Meteorological data from weather stations near the test sites were kindly provided by the Faculty of Wood Science and Technology (Hochschule für Nachhaltige Entwicklung) Eberswalde, German Weather Service (Deutscher Wetterdienst) Potsdam and the Institute of Meteorology of the Free University Berlin.

Laboratory leaching tests were performed according to EN 16105 [11]. Test specimens of about 100 cm^2^ exposed surface area were arranged either in polystyrene containers or in beaker glasses and covered with 25 l/m^2^ water per surface area of the test specimen. Immersion cycles included 1-h immersion, 4-h drying and 1-h immersion. Nine immersion cycles were performed within 3 weeks. The tests were performed at 20 ± 2 °C. The test specimens were dried at a relative humidity of 65 ± 5 % between the immersion events. The eluates of the two immersion events of each immersion cycle were merged and analysed.

### Analysis of runoff samples and eluates

The active substances were analysed in the runoff samples and the eluates from the laboratory tests by ultra-high-performance liquid chromatography coupled with UV detection at BAM, and in runoff samples by liquid chromatography coupled with tandem mass spectrometry at MPA Eberswalde. Intralaboratory precision was determined for eluates from laboratory experiments. Measurement uncertainties were 2–5 % for diuron, 2–7 % for OIT, 2–8 % for terbutryn and 1–6 % for carbendazim depending on the concentration of the analytes. The values were higher at low concentrations that were in the range of the LOQ. Two runoff samples from a field experiment were analysed in both laboratories. The results for diuron, OIT and terbutryn differed by 9, 2 and 10 %, respectively. For further details on the analytical methods, see Schoknecht et al. [8].

## Abbreviations

a.s.: active substance
BPR: biocidal products regulation
CEN: Comité Européen de Normalization
ECHA: European Chemicals Agency
LOQ: limit of quantification
OIT: 2-Octylisothiazol-3(2H)-one
PT: product type
NE: northeast
NW: northwest
SE: southeast
SSW: south-southwest
SW: southwest

## References

[1] Wittmer IK, Bader H-P, Scheidegger R, Singer H, Lück A, Hanke I, Carlsson C, Stamm C (2010). Significance of urban and agricultural land use for biocide and pesticide dynamics in surface waters. Water Res.

[2] Burkhardt M, Zuleeg S, Vonbank R, Schmid P, Hean S, Lamani X, Bester K, Boller M (2011). Leaching of additives from construction materials to urban storm water runoff. Water Sci Technol.

[3] Burkhardt M, Zuleeg S, Vonbank R, Bester K, Carmeliet J, Boller M, Wangler T (2012). Leaching of biocides from façades under natural weather conditions. Environ Sci Technol.

[4] Bollmann UE, Vollertsen J, Carmeliet J, Bester K (2014). Dynamics of biocide emissions from buildings in a suburban stormwater catchment—concentrations, mass loads and emission processes. Water Res.

[5] Gromaire MC, Van de Voorde A, Lorgeoux C, Chebbo G (2015). Benzalkonium runoff from roofs treated with biocide products—in situ pilot-scale study. Water Res.

[6] Wicke D, Matzinger A, Rouault P. Relevanz organischer Spurenstoffe im Regenwasserabfluss Berlins. (2015) Abschlussbericht 2015. http://www.kompetenzwasser.de/fileadmin/user_upload/pdf/forschung/OgRe/Abschlussbericht_OgRe_final_rev2.pdf

[7] Regulation (EU) No 528/2012 of the European Parliament and of the Council of 22 May 2012 concerning the making available on the market and use of biocidal products. (2012) [http://eur-lex.europa.eu/LexUriServ/LexUriServ.do?uri=OJ:L:2012:167:0001:0123:EN:PDF]

[8] Schoknecht U, Mathies H, Wegner R, Uhlig S, Baldauf H, Colson B (2014) Emissions of material preservatives into the environment—realistic estimation of environmental risks through the improved characterisation of the leaching of biocides from treated materials used outdoors. UBA Texte (Real Leach), Report No. UBA-FB 002284, December 2014, Federal Environment Agency (UBA), Dessau-Roßlau, pp. 126 and Report No. UBA-FB 002284/ANL (pp. 127–380)

[9] Blocken B, Derome D, Carmeliet J (2013). Rainwater runoff from building façades: a review. Build Environ.

[10] Schoknecht U, Gruycheva J, Mathies H, Bergmann H, Burkhardt M (2009). Leaching of biocides used in façade coatings under laboratory test conditions. Environ Sci Technol.

[11] EN 16105 (2011) Paints and varnishes—Laboratory method for determination of release of substances from coatings in intermittent contact with water. CEN/TC 139:2011. CEN- CENELEC Management Centre Brussels

[12] Styszko K, Bollmann UE, Bester K (2015). Leaching of biocides from polymer renders under wet/dry cycles—Rates and mechanisms. Chemosphere.

[13] NT build 509 (2005) NORDTEST Method Leaching of active ingredients from preservative-treated timber—Semi-field testing. Nordic Innovation Centre

[14] CEN/TR 16663 (2014) Durability of wood and wood-based products - Determination of emissions from preservative treated wood to the environment—Wooden commodities exposed in Use Class 3 (Not covered, not in contact with the ground)—Semi-field method. CEN/TC 38:2014. CEN-CENELEC Management Centre Brussels

[15] Guidance on a semi-field test method for materials that are treated with biocides. Report No. UBA-FB 002284, December 2014, Federal Environment Agency (UBA), Dessau- Roßlau, pp. 126 and Report No. UBA-FB 002284/ANL (pp. 127 – 380), publication at ECHA website in preparation

[16] Guidance on a laboratory leaching test method for materials that are treated with biocides. Report No. UBA-FB 002284, December 2014, Federal Environment Agency (UBA), Dessau- Roßlau, pp. 126 and Report No. UBA-FB 002284/ANL (pp. 127 – 380), publication at ECHA website in preparation

[17] Tomlin C (2009). The Pesticide Manual.

[18] Paulus W (2005). Directory of Microbicides for the Protection of Materials—A Handbook.

[19] DIN 4108-3 (2014) Thermal protection and energy economy in buildings—Part 3: Protection against moisture subject to climate conditions—Requirements and directions for design and construction

